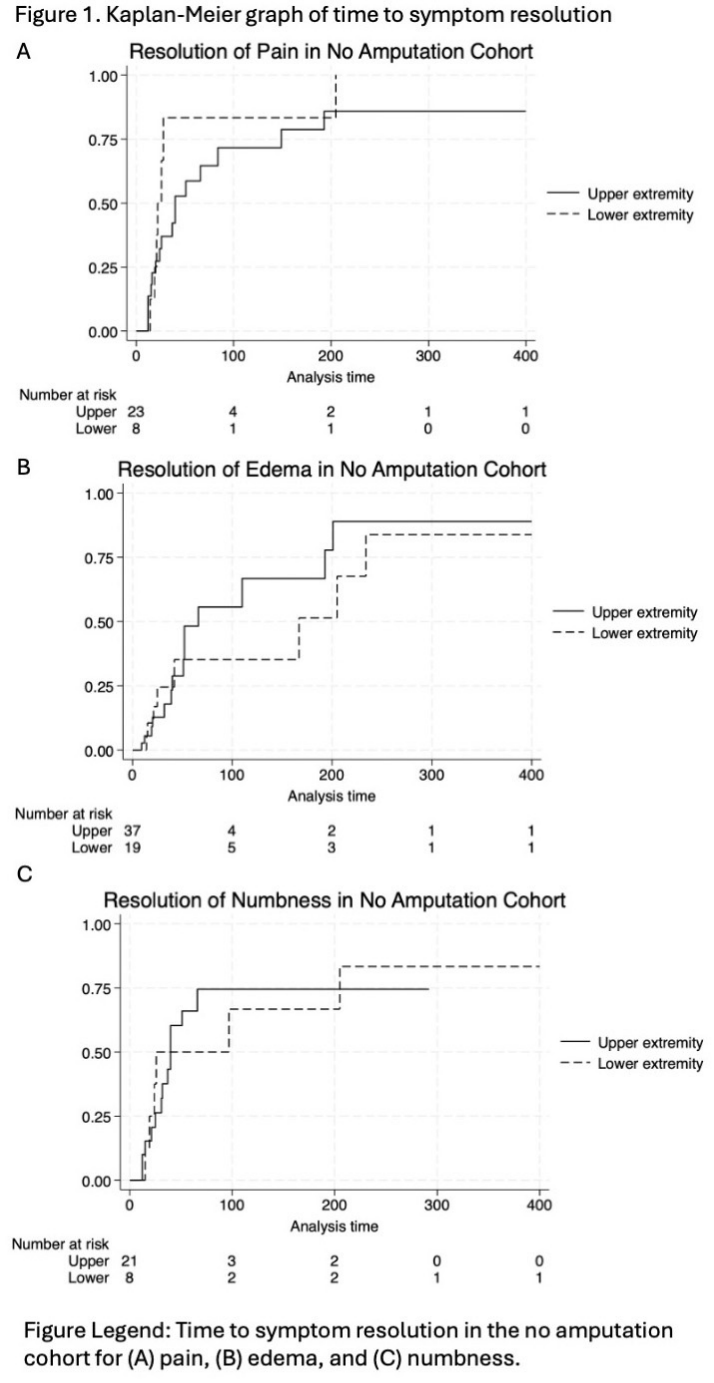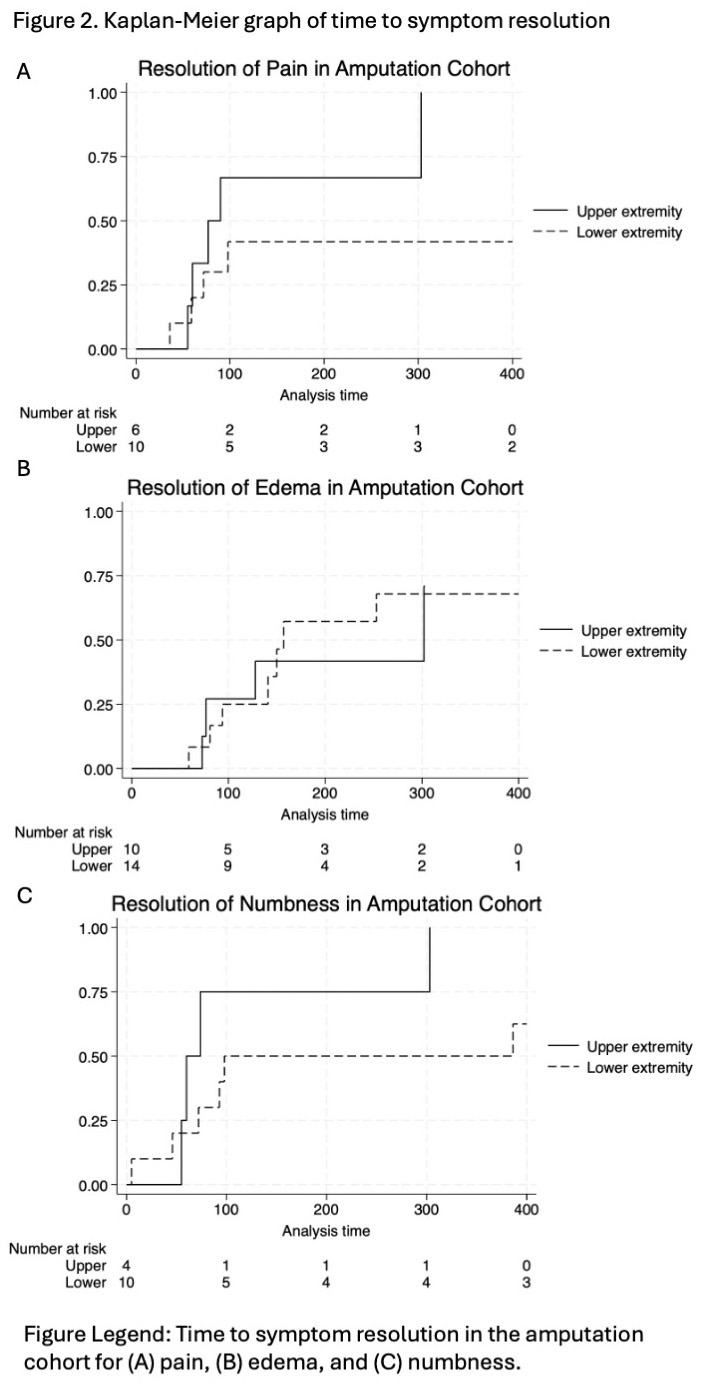# 995 Amputation and Extremity-Specific Outcomes in Frostbite: A Comparative Longitudinal Study

**DOI:** 10.1093/jbcr/iraf019.526

**Published:** 2025-04-01

**Authors:** Lexy Kindt, Charly Vang, Emily Colonna, Rediat Tilahun, Sarah Zemek, Lisa Nelson, Jana Almendinger, Patricia White, Kyle Schmitz, Derek Lumbard, Rachel Nygaard

**Affiliations:** Hennepin County Medical Center; Hennepin Health Care Research Institute; Hennepin Healthcare; Hennepin Healthcare Research Institute; Hennepin Healthcare; Hennepin Healthcare; Hennepin County Medical Center; Hennepin County Medical Center; Hennepin Healthcare; Hennepin Healthcare; Hennepin Healthcare

## Abstract

**Introduction:**

Severe frostbite injury can lead to significant long-term functional impairments. This study aims to assess long-term function and symptom resolution in upper vs. lower extremity frostbite. We hypothesize that symptom resolution is slower in upper extremity injury and need for functional assistance is higher in lower extremity injury.

**Methods:**

A longitudinal cohort of 182 severe frostbite injured patients, defined with a post-rewarming perfusion deficit on imaging and/or clinical diagnosis, included 137 patients with isolated upper or lower extremity injury. Analyses were stratified by amputation and only included those with documented symptoms (neurogenic pain, edema, and numbness) or need for functional assistance (aid with activities of daily living (ADLs) or ambulation) at discharge. Logistic regression was performed to assess need for functional assistance and Cox regression analyzed time to symptom resolution.

**Results:**

There were equal distribution between upper extremity (51%) and lower extremity (49%) frostbite injuries. Most upper (61%) and lower (66%) extremity injured patients needed assistance at discharge. The overall rate of amputation was 27%, (33% lower and 21% upper, p = 0.133). In patients without amputations, logistic regression showed no significant difference in functional assistance need between upper and lower extremity injuries (OR 1.32, p=0.709). However, among those with amputations, lower extremity injuries were associated with significantly worse functional outcomes (OR 21.67, p=0.008). Cox regression showed non-significant differences in time to symptom resolution between upper and lower extremity injuries in non-amputees (fig 1) and amputees (fig 2).

**Conclusions:**

This study offers a novel analysis of extremity-specific outcomes, providing new insights into functional recovery and symptom resolution in the largest contemporary cohort of severe frostbite patients. Lower extremity amputees required more functional assistance, while no significant differences in symptom resolution were observed between upper and lower extremity injuries. These findings can guide tailored rehabilitation strategies, enhance functional outcomes, and improve resource allocation in burn care settings. Future research should explore whether limited access to assistive devices, particularly for upper extremity amputees, contributes to differing outcomes.

**Applicability of Research to Practice:**

Collaborative efforts to delineate access to assistive-devices and define standard symptom measurements in frostbite injuries may help improve functional outcomes in this high need, low resourced population.

**Funding for the Study:**

N/A